# Effects of cavity depth (moderate vs. deep with pulp exposure) on the release of prostaglandin E2 and nitric oxide in rat mandibular incisors

**DOI:** 10.3389/fdmed.2025.1671128

**Published:** 2025-11-03

**Authors:** Hassanien Riyadh, Anas F. Mahdee

**Affiliations:** ^1^ MSC student in the Department of Restorative and Aesthetic Dentistry, College of Dentistry, University of Baghdad, Baghdad, Iraq; ^2^ Professor in the Department of Restorative and Aesthetic Dentistry, College of Dentistry, University of Baghdad, Baghdad, Iraq

**Keywords:** pulp inflammation, cavity depth, rat teeth, cavity preparation, pulp exposure, ELISA

## Abstract

**Background/objectives:**

Inflammatory mediators such as prostaglandin E2 (PGE2) and nitric oxide (NO) are key indicators of pulp response to mechanical trauma. However, the influence of cavity depth on their release dynamics remains unclear. This study aimed to evaluate the effects of different cavity depths—moderate (without pulp exposure) and deep (with pulp exposure)—on the release of PGE2 and NO in the pulp tissue of rat mandibular incisors at two time intervals (3 and 9 h).

**Methods:**

In total, 40 male Wistar rats were divided into two main groups (*n* = 20) based on cavity depth. A split-mouth design was used, with cavities of different depths prepared on the left mandibular incisors, leaving the right incisors without cavities as controls. All the prepared cavities were sealed with glass ionomer filling until 3 or 9 h (*n* = 10), when pulp tissue was removed. Homogenates were prepared and analyzed by ELISA for PGE2 and NO levels.

**Results:**

Cavities without pulp exposure elicited 2.1-fold higher PGE2 (median: 3.44 vs. 1.81 ng/mL; *p* < 0.001) and 1.3-fold higher NO (median: 55.45 vs. 43.76 μmol/L; *p* < 0.01) compared to exposed cavities at 3 h—a paradoxical finding suggesting intact pulp architecture potentiates acute inflammatory signaling. This disparity persisted at 9 h (PGE2: 3.18 vs. 1.81 ng/mL; NO: 49.94 vs. 44.98 μmol/L), though with attenuated significance (*p* < 0.05).

**Conclusion:**

Cavity preparation induces an early inflammatory response in the pulp, as indicated by increased PGE2 and NO levels. The inflammatory response was more pronounced in cavities without pulp exposure, suggesting that maintaining pulp integrity favors a regulated inflammatory response conducive to healing, while exposure may promote irreversible damage.

## 1 Introduction

The dental pulp is a specialized connective tissue encased within hard dentin, making it particularly vulnerable to injury from mechanical, thermal, and chemical stimuli during restorative dental procedures. This confined condition of the pulp means that even slight inflammatory incidents can quickly intensify, jeopardizing the pulp's vitality and resulting in irreversible injury (1, 2).

Cavity preparation is one of the most prevalent iatrogenic factors affecting the pulp. The extent of mechanical damage during cavity preparation—specifically, the depth of the preparation into the pulp chamber—significantly influences the intensity of the inflammatory reaction. Conservative procedures that retain a significant amount of dentin may preserve the pulp by reducing the transmission of noxious stimuli. Conversely, extensive preparations, particularly those leading to pulp exposure, can trigger severe inflammatory responses that render the pulp susceptible to necrosis (3, 4).

Prostaglandin E2 (PGE2) and nitric oxide (NO) are primary inflammatory mediators implicated in the initial stages of pulpal inflammation. PGE2, synthesized through the cyclooxygenase pathway, is recognized for its strong vasodilatory and nociceptive properties, increasing vascular permeability and sensitizing nociceptors to pain stimuli. Nitric oxide, predominantly produced by inducible nitric oxide synthase (iNOS) in inflamed tissues, also has a role in vascular control and the recruitment of immune cells. The levels of both molecules promptly increase after tissue injury and they are regarded as key contributors to the development of pulpitis (5, 6).

The rat mandibular incisor was selected for this study due to its well-established utility in pulp biology research. Its continuous growth and standardized anatomy facilitate reproducible experimental interventions, while its neurovascular and inflammatory responses closely mirror early human pulpitis dynamics (7). Notably, metabolic and inflammatory reactions in rats occur at an accelerated rate compared to humans; peak PGE2 and NO levels are typically observed within 3–12 h postinjury, justifying our focus on the 3- and 9-h time points (8). These intervals capture the acute phase of mediator release, avoiding the confounding effects of chronic degeneration or repair.

While the roles of PGE2 and NO in pulpitis are established, their quantitative modulation by mechanical injury depth—specifically, the differential effects of moderate (non-exposing) vs. deep (pulp-exposing) cavities—remains uncharacterized. Studies in the literature have focused primarily on chemical or microbial stimuli, creating a critical knowledge gap regarding iatrogenic mechanical injury gradients (9, 10).

We hypothesized that deep cavities would trigger a higher level of mediator release than moderate cavities. This study aimed to evaluate the effects of moderate and deep cavity preparations (with and without pulp exposure) on the release of PGE2 and NO at 3 and 9 h postinjury intervals within the pulp tissue of rat mandibular incisors.

## 2 Materials and methods

### 2.1 Sample selection

This study followed the Preferred Reporting Items for Animal Studies in Endodontology (PRIASE) guidelines for animal research in endodontology. All the procedures were approved by the Ethical Committee of the University of Baghdad (Protocol #902524, 22 February 2024). In total, 40 male Wistar rats (6–8 weeks old) were selected in this study and allocated using a pregenerated random number table (Fisher and Yates, 1948) with sealed envelope concealment. The experimental model was a split-mouth design, which involved preparing cavities with different depths on the left mandibular incisors, leaving the right incisors without cavities as controls. The animals were divided into two main groups (*n* = 20) according to the cavity depth, namely, moderate depth cavities without pulp exposure and deep cavities with pulp exposure. These two groups were further subdivided according to time point (*n* = 10) into the early (3 h) and delayed (9 h) groups. The sample size (*n* = 10/group) was chosen to match previous rat pulp studies that detected inflammatory mediators (*n* = 8–12/group for PGE2/NO measurements), ensuring adequate power for our primary outcome comparisons while minimizing animal use (10).

### 2.2 Sample preparation

**Figure 1:**
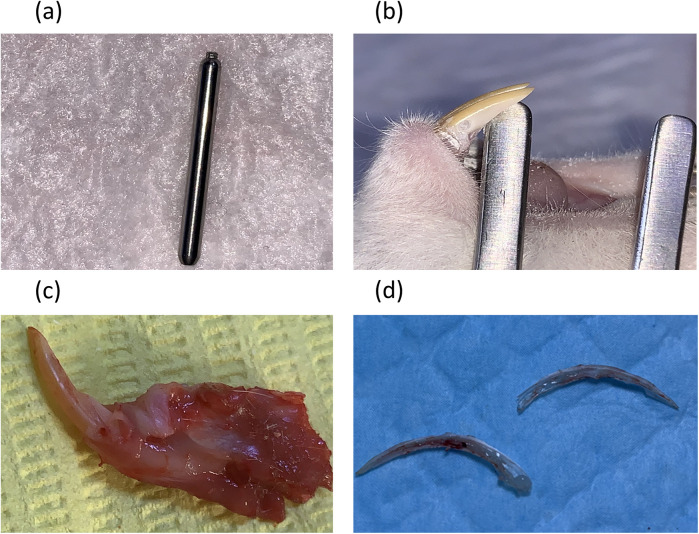
Cavity preparation and teeth dissection procedures. **(a)** A Diamond Bur Komet® PrepMarker® instrument (DM05) was used to prepare the cavities. **(b)** A non-exposure cavity. **(c)** A cavity with pulp exposure. **(d)** The pulp after vertical splitting of the mandibular incisor.

The levels of PGE2 and NO were measured in the pulp tissue after animal euthanasia and pulp tissue extractions. The rats were anesthetized using an intramuscular injection of ketamine (50 mg/kg) and xylazine (5 mg/kg), dosed according to the individual’s body weight (measured prior to the procedure). The depth of anesthesia was confirmed by the absence of a pedal reflex. The cavities were cut using a Diamond Bur Komet® PrepMarker® instrument (DM05), which features a 0.9 mm diameter and a 0.5 mm cutting length before reaching the non-cutting shank. This prevented penetration to further depths, which allowed us to use the full depth of the bur to create a reproducible cavity with a controlled depth and dimensions. This cavity exposed pulp in the most cervical part of the mandibular incisor after removing the gingiva using a sterile scalpel blade. Bleeding at the exposure site was controlled by pressing a sterile saline-soaked cotton pellet over the site for 1–2 min (Figure 1). The cavity was then sealed using glass ionomer filling cement (Medifil, ProMedica, OH, USA) without any capping material. The cavities in the group without pulp exposure were made on the distal side of the mandibular incisor teeth using the same Komet® PrepMarker® instrument (11). After 3 or 9 h, the animals were euthanized by cervical dislocation before surgical extraction of the mandibular incisors. Each tooth was sectioned longitudinally under a magnifying binocular loupes (Jucheng, China) by making grooves on the dentin without exposing the pulp using a cutting disc with water cooling. The teeth were then separated into two halves using surgical blades and immediately rinsed with RNase/DNase-free PBS (ThermoFisher, USA) to prevent tissue contamination before extracting the pulp tissue with a sterile tweezer (Figure 1d).

Following extraction, each pulp tissue sample was immediately placed into a preweighed Eppendorf tube containing 0.5 mL of ice-cold phosphate-buffered saline (PBS). The time from animal euthanasia to tissue immersion in PBS was rigorously maintained at less than 5 min per sample to prevent mediator degradation.

Homogenization was performed mechanically in an ice bath to maintain a temperature of 4 °C throughout the process. The homogenized samples were then stored overnight at 4 °C to allow for complete protein extraction. The following day, the samples were centrifuged at 5,000 rpm for 20 min in a refrigerated centrifuge (Eppendorf 5417 R, Germany) at 4 °C. The resulting supernatant was carefully aliquoted and immediately stored at −80 °C until assayed. This strict cold chain protocol ensured the stability of the heat-labile inflammatory mediators PGE2 and NO. PGE2 (Biont, China) and NO (Biont, China) ELISA kits were used to detect the levels of these two biomarkers according to their manufacturer's instructions at a wavelength of 450 ± 10 nm.

### 2.3 Statistical analysis

The statistical analysis was performed using IBM SPSS Statistics for Windows, Version 28.0 (Released 2021; IBM Corp., Armonk, NY, USA). The Shapiro–Wilk test was used to determine the normality of the results at *p* ≤ 0.05. A multivariate analysis was used to identify the effects of tooth type (control vs. cavity), depth of cavity (without vs. with exposure), and time (3 h vs. 9 h) on the levels of PGE2 and NO. This approach was preferred over a traditional ANOVA due to its capacity to handle repeated measures, missing data, and interanimal variation while preserving statistical power. Furthermore, the Kruskal–Wallis test was used to compare the levels of these markers at *p* ≤ 0.05 between the controls (*n* = 20) and both cavity type groups (*n* = 10 for each) at each time point. In addition, Spearman’s correlation assay was used to identify the relationship between the two biomarkers (PGE2 and NO). We calculated the effect sizes and confidence intervals to quantify the magnitude and precision of the observed differences. For the between-group comparisons (e.g., non-exposed vs. exposed), the non-parametric *r* effect size was derived from the Z-statistic of the Mann–Whitney *U*-test: *r* = *Z*/√*N*, where *N* is the total number of observations. The effect sizes were interpreted as follows: *r* = 0.10 was small, 0.30 was medium, and 0.50 was large. The common language effect size (CLES) was calculated to indicate the probability that a randomly selected score from one group would be greater than a score from another group. Furthermore, the 95% confidence intervals (CIs) for the medians were estimated using bootstrapping with 5,000 resamples. All the effect sizes and CIs were calculated using the “rstatix” and “boot” packages in R software (version 4.3.1), respectively. A *post hoc* power analysis based on the observed effect size for the primary outcome (PGE2 level) indicated a power of 92% (*α*=0.05, *n* = 10/group), reducing the risk of Type II error for this key comparison.

## 3 Results

The collected data were found not to be normally distributed in the Shapiro–Wilk test (*p *˃ 0.05); therefore, all the concentration measurements of PEG2 and NO are presented as a median and interquartile range (Table 1). The medians for both the PGE2 and NO concentrations were higher within the cavity groups in comparison to the controls. Moreover, the medians for these biomarkers were higher in the groups with cavities without pulp exposure than in those with pulp exposure. The concentrations of PGE2 and NO after 9 h were slightly decreased compared with their values after 3 h for all groups, except for NO concentration in the cavity with pulp exposure group, in which the concentration was slightly higher.

**Table 1:** Descriptive statistics showing medians and interquartile ranges for concentrations of PGE2 (ng/mL) and NO (µmol/L) in all the groups in the study.

Group	Time (h)	Median	PGE2 (ng/mL)		Median	NO (µmol/L)	
			95% CI for median	Interquartile range		95% CI for median	Interquartile range
Control	3	1.805	[1.512, 2.101]	0.896	41.375	[38.921, 44.102]	8.746
Non-exposed cavity		3.436***	[2.987, 3.955]	1.027	55.451**	[52.110, 58.900]	11.212
Cavity with pulp exposure		2.248*	[1.852, 2.751]	1.052	43.760	[41.002, 46.505]	8.070
Control	9	1.396	[1.201, 1.655]	0.522	41.069	[38.554, 43.888]	8.458
Non-exposed cavity		3.182**	[2.512, 4.105]	2.495	49.939*	[46.852, 53.101]	9.938
Cavity with pulp exposure		1.808	[1.552, 2.101]	0.378	44.979	[42.105, 47.852]	7.999

* *p* < 0.05; **p < 0.01; and ***p < 0.001.

**Figure 2:**
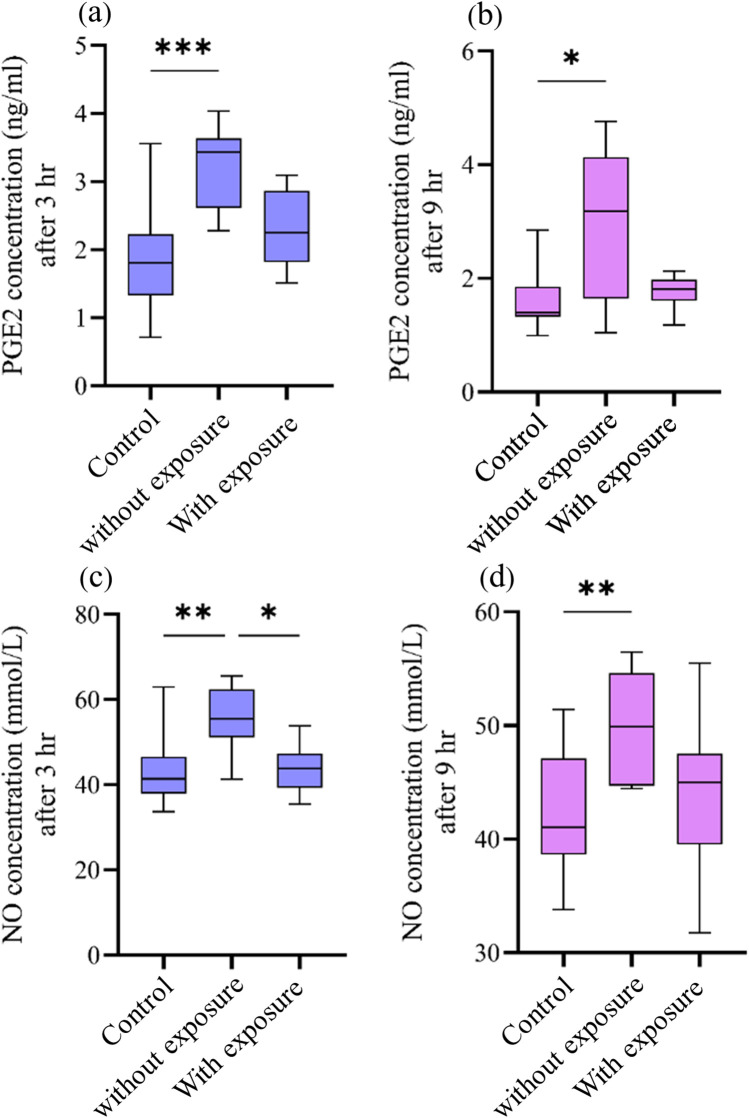
Effect of cavity preparation on PGE2 (ng/mL) and NO (µmol/L) concentrations between the controls and the groups with cavities without and with pulp exposure at two time points. The box plots represent median value (horizontal line), interquartile range (box), and the minimum and maximum values (vertical lines). **(a,b)** The PGE2 concentrations in the control group, non-exposed cavity group, and cavity with pulp exposure group, after 3 h and 9 h, respectively. **(c,d)** The concentration of NO in the same groups after 3 h and 9 h, respectively. Statistical significant difference in the multiple comparison test is represented by * for *p* ≤ 0.05, ** for *p* ≤ 0.01, and *** for *p* ≤ 0.001.

The multivariate mixed model analysis showed that tooth type (control vs. cavity) and cavity depth (without vs. with pulp exposure) had statistically significant effects (*p* ≤ 0.001) on the PGE2 and NO concentrations. However, the time points had no statistically significant effect (*p* ˃ 0.05) (Table 2). To identify any significant differences in the concentrations of PGE2 and NO between controls and the two types of cavity groups, the Kruskal–Wallis test was used. This test found statistically significant differences (*p* ≤ 0.05) between all the tested groups at both time points. The results of the multiple comparison tests for PGE2 and NO at each time point are presented in Figure 2. For PGE2 concentration, only the group with cavities without pulp exposure showed statistically significant differences in comparison to their controls at both 3 h (*p* ≤ 0.001) and 9 h (*p* ≤ 0.05) (Figures 2a,b, respectively). Similarly, NO concentration was also statistically significantly different in the group with cavities without pulp exposure compared to their controls at the 3-h time interval (*p* ≤ 0.01) (Figures 2c,d). Furthermore, a statistically significant difference (*p* ≤ 0.05) was only found between the groups without and with pulp exposure at the 3-h time point. Spearman's correlation analysis revealed a statistically significant positive correlation between the PGE2 and NO concentrations at both time points. At 3 h, a moderate correlation was observed (*r* = 0.507, *p* = 0.02), which became stronger at 9 h (*r* = 0.602, *p* = 0.005), indicating that an increase in one mediator tends to be associated with a concurrent increase in the other (Table 3).

**Table 2:** Mixed-effect multivariate analysis of the effect of the different variables in the study on the concentrations of PGE2 (ng/mL) and NO (µmol/L) in all the groups.

Parameter^a^	PGE2		NO	
	t	*p*-Value	t	*p*-Value
Tooth (control vs. cavity)	−4.702	0.000	−4.756	0.000
Time (3 h vs. 9 h)	1.574	0.124	0.828	0.413
Cavity depth (non-exposure vs. with exposure)	4.034	0.000	4.712	0.000

^a^ Results from a linear mixed-effects model with rat ID as the random intercept.

**Table 3:** Spearman's correlation analysis of PGE2 and NO at the two time points in the study (3 and 9 h).

Time point	Spearman's R	*P*-Value	NO
PGE2	3 h	*R*	0.507
		*p*-Value	0.02
	9 h	*R*	0.602
		*p*-Value	0.005

## 4 Discussion

This study found that cavities cause higher levels of two biomarkers (PGE2 and NO) within the dental pulp, indicating an early inflammatory response. This effect was found to be dependent on the depth of the cavity rather than the time of observation (3 and 9 h). Both the PGE2 and NO pathways are believed to play important roles in the regulation of normal physiological processes, inflammation, and pathogenesis of pulp diseases (12). A deeper understanding of these processes can assist in clinical procedures by suggesting the best therapeutic protocols, particularly for vital pulp therapy, leading to optimized treatment outcomes. To the best of the authors’ knowledge, this is the first study that directly assesses the impact of cavity preparation and its depth on PGE2 and NO release from dental pulp homogenates.

Prostaglandin E2 is a crucial modulator of pulp inflammation, increasing vascular permeability, facilitating vasodilation, and sensitizing nociceptors, which results in pain perception (2). PGE2 also sensitizes peripheral nociceptors, thereby reducing the pain threshold and facilitating the onset of hyperalgesia in inflamed pulp tissue (4). Nonetheless, high or sustained release of PGE2 may lead to chronic inflammation and pulpal necrosis (5). The increased levels of PGE2 observed in this study after cavity preparation further substantiate its essential role in triggering the inflammatory response after mechanical injury. Likewise, nitric oxide serves as a multifunctional signaling molecule that facilitates vasodilation, recruits inflammatory cells, and modulates immunological responses. NO also serves a dual function in the advancement of pulp inflammation. In the initial inflammatory phase (0–3 h), NO exerts protective effects through multiple mechanisms. First, it promotes vasodilation by activating soluble guanylate cyclase (sGC), resulting in elevated intracellular cyclic GMP, which facilitates the relaxation of the pulp microvasculature and enhances blood perfusion. Furthermore, NO enhances the innate immune system by generating peroxynitrite (ONOO⁻) upon reaction with superoxide (O₂), demonstrating antibacterial properties. It also temporarily inhibits proinflammatory cytokines such as TNF-α and IL-1β, hence regulating excessive inflammation (6). This study found that the pulp from non-exposed cavities had increased NO levels at 3 h of 55.45 µmol/L in contrast to 41.38 µmol/L in the controls, suggesting vasodilation-mediated regulation of the acute inflammatory response.

Conversely, in the late inflammatory phase (>6 h), NO becomes increasingly destructive. Increased nitric oxide levels interact with O₂ to generate elevated concentrations of peroxynitrite, which can initiate oxidative stress reactions such as lipid peroxidation, damage to odontoblast membranes, and DNA strand breaks, resulting in fibroblast apoptosis. Nitric oxide also augments TRPV1 channel activity, facilitating pain sensitization. High NO levels act as a compensatory mechanism by suppressing inducible nitric oxide synthase (iNOS) expression over time, which may result in a decrease in NO production (13). This trend is supported by the mild rise of 8.2% detected in the pulp from exposed cavities at 9 h compared to that at 3 h.

Clinically, early NO level elevation correlates with positive results in reversible pulpitis, whereas a sustained increase in the later phase may indicate irreversible damage and necrosis, frequently necessitating root canal treatment. Targeted manipulation of NO pathways may provide therapeutic advantages: the early administration of L-NAME (an NOS inhibitor) could mitigate excessive vasodilation, while antioxidants such as N-acetylcysteine (NAC) may counteract surplus peroxynitrite in the latter stages (14, 15).

The presence and regulation of these mediators dictate both the severity of the inflammatory response and the potential for pulp healing or irreversible injury. Understanding their behavior in reaction to various injury types (with or without pulp exposure) may assist clinicians in choosing restorative methods that reduce adverse inflammatory reactions and facilitate healing, while conserving pulp vitality wherever feasible.

The findings of this study are in agreement with the literature (16–18), as PGE2 and NO secretion increased following trauma or injury to the pulp. However, this study has revealed a paradigm-shifting observation regarding pulp inflammatory dynamics.

The “non-exposure paradox,” where structurally intact pulp (i.e., from non-exposed cavities) has higher inflammatory mediator release than severely injured pulp (i.e., from exposed cavities), was demonstrated in this study as cavities stopping short of pulp exposure generated 96.3% higher PGE2 and 34.1% higher NO compared to the controls at 3 h (*p* < 0.001), challenging the classical assumption that greater injury elicits stronger inflammation (19).

Furthermore, the significant difference observed between the two cavity types at 3 h, but not at 9 h, suggests that cavity depth plays a more prominent role in modulating the immediate inflammatory response rather than the delayed response. The more pronounced mediator release in the early stage could be attributed to the initial mechanical stimulus and subsequent activation of the COX-2 and iNOS pathways (16, 17).

As mentioned above, this study’s results showed that pulp from cavities without pulp exposure had higher median concentrations of both PGE2 and NO compared to pulp from cavities with pulp exposure. This observation suggests a focused immune response in structurally intact pulp. This was demonstrated by Bletsa et al. (7), who found that preserved odontoblast layers in non-exposed cavities likely maintain paracrine signaling (e.g., TGF-β1 and CGRP) that amplifies early mediator release while preventing necrosis. In contrast, the decrease in mediator release in the pulp from teeth with pulp exposure may indicate an impairment of the cellular components generating PGE2 and NO or a mere cessation of the signal due to tissue necrosis (3). This study additionally found that the levels of PGE2 and NO did not exhibit significant differences between the two time intervals (3 h vs. 9 h). This suggests that the peak response of the inflammatory mediators commenced shortly after injury (i.e., <3 h) and remained consistent during the initial hours, supporting previous research indicating that PGE2 and NO are among the earliest molecules upregulated in the acute inflammatory phase (10). This sustained increase may facilitate vasodilation, immune cell recruitment, and local tissue perfusion, thereby promoting a regulated inflammatory environment. Nonetheless, the extended presence of such mediators, particularly beyond the acute phase, may facilitate persistent nociceptor sensitization and pain perception. It is commonly believed that these levels diminish as healing progresses, assuming the injury is not aggravated or becomes infected; otherwise, their persistence may lead to chronic inflammation and pulpal degeneration (20).

Furthermore, it appears that these mediators are coregulated during the early inflammatory response because of the positive correlation between PGE2 and NO at both 3 and 9 h. Their simultaneous increase suggests a common upstream signaling pathway, which may be triggered by NF-κB activation or the production of cytokines such as TNF-α and IL-1β. This interaction may increase pain sensitivity and the local inflammatory response, but it may also help coordinate the healing process by boosting immune and vascular cell activity. Understanding this relationship may help clinicians develop treatment plans that target simultaneous modulation of both mediators to successfully manage pain and inflammation (16).

These findings offer direct experimental justification for current minimally invasive caries management approaches. First, selective caries removal—preserving as little as 0.25–0.5 mm of residual dentin—appears sufficient to maintain a pulp architecture that enables a coordinated immune response. This aligns with clinical outcomes that show greater success rates in vital pulp therapy when residual dentin or dentin bridges are preserved (21). Second, the marked reduction in PGE2 levels observed in the exposed cavity group vs. the non-exposed cavity group (approximately 53%) underscores the risk associated with overpreparation. Excessive removal of dentin may impair reparative signaling and increase susceptibility to pulpal necrosis. Finally, the observed peak in the inflammatory mediators at the 3-h interval supports the concept of an early therapeutic window. Interventions such as pulp capping or anti-inflammatory therapy may be most effective when applied promptly following injury, ideally within the first few hours (22). These insights reinforce the principles of biological caries removal as advocated by the FDI (World Dental Federation) guidelines, especially for young permanent teeth with high regenerative capacity (23).

It is important to acknowledge that the use of rat mandibular incisors presents inherent limitations when extrapolating the findings to human clinical scenarios. Unlike human molars, rat incisors exhibit continuous eruption and possess thinner dentin walls, which may influence both the diffusion of inflammatory mediators and the mechanical response to cavity preparation (24). While the rat model offers valuable insights into pulp biology and inflammatory dynamics, caution must be exercised when generalizing the magnitude and timing of the pulp responses observed in this study to human teeth (25). Future validation in human tissue or extracted molars with standardized cavity preparations is warranted to confirm clinical applicability. A key limitation is the absence of a histological confirmation of two critical aspects: the exact cavity dimensions and the pulp's vital status in each group. While the use of a depth-limited bur promotes reproducibility and the differential mediator levels imply a gradient of vitality, future work incorporating histology is needed to directly validate these findings. Furthermore, this study concentrated on the early postinjury intervals (3 and 9 h), because the levels of the selected markers are significant within this period of inflammation (10). However, understanding their levels after this stage is also recommended. In addition, while the ELISA testing procedure yields quantitative data regarding the mediators’ levels, it cannot provide a spatial localization of their expression within the pulp tissue (9). Therefore, specific identification of the pulp cells responsible for the secretion of these markers should be considered.

## 5 Conclusions

This study emphasizes the critical influence of cavity depth on the inflammatory response of dental pulp, as indicated by PGE2 and NO levels in a rat model. Three principal conclusions were derived from the analysis.

First, cavities that maintained pulp integrity resulted in significantly elevated levels of inflammatory mediators, with a 96% rise in PGE2 and a 34% increase in NO compared to controls (*p* < 0.001), suggesting that structurally intact pulp generates a more coordinated and vigorous immune response. This observation defies the common belief that increased tissue damage results in stronger inflammation, leading to what may be referred to as the “pulp integrity paradox.”

Second, direct pulp exposure resulted in a 35% decrease in PGE2 release compared to non-exposed cavities (*p* < 0.05), possibly attributable to necrosis-induced impairment of odontoblast function and the disintegration of paracrine signaling networks. This indicates that overt damage may hinder, rather than enhance, mediator production.

Finally, the temporal analysis demonstrated dynamic NO behavior, with an initial peak at 3 h (55.45 µmol/L) followed by a relative decrease at 9 h. This biphasic pattern may indicate a transition from protective vasodilation in the initial phase—potentially facilitated by eNOS—to a cytotoxic condition characterized by the peroxynitrite and iNOS pathways in the later phase. These findings provide insight into possible therapeutic opportunities for clinical intervention.

Clinically, these findings support the use of less invasive restorative procedures, including selective caries removal, to maintain the vitality of the pulp and any remaining dentin.

## Data Availability

The original contributions presented in the study are included in the article/Supplementary Material, further inquiries can be directed to the corresponding author.
